# Skin Lesion Analysis and Cancer Detection Based on Machine/Deep Learning Techniques: A Comprehensive Survey

**DOI:** 10.3390/life13010146

**Published:** 2023-01-04

**Authors:** Mehwish Zafar, Muhammad Imran Sharif, Muhammad Irfan Sharif, Seifedine Kadry, Syed Ahmad Chan Bukhari, Hafiz Tayyab Rauf

**Affiliations:** 1 Department of Computer Science, COMSATS University Islamabad, Wah Campus, Wah Cantt 47040, Pakistan; mehvishzafar999@gmail.com (M.Z.); mimrannsharif@gmail.com (M.I.S.); 2 Department of Computer Science, University of Education, Jauharabad Campus, Khushāb 41200, Pakistan; muhammadirfaansharif@gmail.com; 3 Department of Applied Data Science, Noroff University College, 4612 Kristiansand, Norway; 4 Artificial Intelligence Research Center (AIRC), Ajman University, Ajman P.O. Box 346, United Arab Emirates; 5 Department of Electrical and Computer Engineering, Lebanese American University, Byblos P.O. Box 13-5053, Lebanon; 6 Division of Computer Science, Mathematics and Science, Collins College of Professional Studies, St. John’s University, Queens, NY 11439, USA; bukharis@stjohns.edu; 7 Independent Researcher, Bradford BD8 0HS, UK; hafiztayyabrauf093@gmail.com

**Keywords:** skin cancer, machine learning, deep learning, segmentation, melanoma, classification

## Abstract

The skin is the human body’s largest organ and its cancer is considered among the most dangerous kinds of cancer. Various pathological variations in the human body can cause abnormal cell growth due to genetic disorders. These changes in human skin cells are very dangerous. Skin cancer slowly develops over further parts of the body and because of the high mortality rate of skin cancer, early diagnosis is essential. The visual checkup and the manual examination of the skin lesions are very tricky for the determination of skin cancer. Considering these concerns, numerous early recognition approaches have been proposed for skin cancer. With the fast progression in computer-aided diagnosis systems, a variety of deep learning, machine learning, and computer vision approaches were merged for the determination of medical samples and uncommon skin lesion samples. This research provides an extensive literature review of the methodologies, techniques, and approaches applied for the examination of skin lesions to date. This survey includes preprocessing, segmentation, feature extraction, selection, and classification approaches for skin cancer recognition. The results of these approaches are very impressive but still, some challenges occur in the analysis of skin lesions because of complex and rare features. Hence, the main objective is to examine the existing techniques utilized in the discovery of skin cancer by finding the obstacle that helps researchers contribute to future research.

## 1. Introduction

For a better understanding of skin cancer, clinicians should require a basic awareness of the skin. There are three main layers epidermis, dermis, and subcutaneous fat [1]. Skin cancer is the state when the uncommon development of the skin cell becomes uncontrolled [2]. Every day some old skin cells die, and new cells take their place. However, when this process takes the wrong direction, a situation occurs when the old cells are not in the dying stage but become dead and new cells grow when there is no need for them. The extra number of skin cells creates a mass of tissue and becomes a tumor [3,4]. For the enhancement of the diagnostic process, dermoscopy has begun; itis a non-invasive approach that gets illuminated and enhanced images of skin spots. This approach is utilized by the dermatologist for skin cancer recognition, traditionally performed by visual inspection and manual screening which is not accurate and also time-consuming [5]. However, the improvement has been made on account of the latest approaches of machine learning for the timely recognition of fatal cancerous diseases. Some skin tumors are benign and curable if detected at the early stage, so they hardly turn into cancer. Melanoma is a very serious and dangerous category of skin tumor. There are further numerous divisions of skin tumors and each cancer type depends upon the behavior of the abnormal cells. There are the following categories of skin cancer melanoma [6], squamous and basal cell carcinoma (together called nonmelanocytic skin cancers [7] and Merkel skin carcinoma [8]. Different types of changes occur in the human body as skin cancer has several symptoms. The changes that happen are color fadedness of the skin, an increase in the size of the mole, and ulceration. However, these symptoms can vary from type to type. Usually, the symptoms of basal cell carcinoma (BCC) include a swollen smooth surface on the shoulder and neck and blood vessels becoming visible in a tumor. These tumors are developed with the bleeding from the center point of the tumor, but these types of cancer are treated completely. In squamous cell carcinoma (SCC) cancer shows in the form of nodules that look hard in presence. It can result in bleeding from the tumor and ulceration. If this type of cancer is not treated immediately a large mass appearance occurs on the skin. Merkel skin carcinoma (MCC) cancer presents as a painless red spot so it is taken as a cyst [9]. UV is a serious revisable harmful agent for skin cancer and others are environmentally affected skin disorders. UV is also known as a big source of Vitamin D; therefore, UV has a mixed influence on human health. Excessive intake of UV can cause health risks such as malignancy, pigmentary modification, wrinkling, and atrophy. The UV molecularly and epidemiologically has an association with different categories of skin cancers. The genetic factor becomes another element that may lead to skin disorders [10]. As it is known that the ratio of skin diseases increases every year, it is essential to take precautionary measures to avoid such disorders. UV rays are a familiar environmental source of skin cancer so protection of the skin from these rays is necessary. The measure that one should take to avoid UV rays is to avoid direct exposure to mid-day sun, and textile protection by wearing appropriate clothing and by using sunscreen. It is essential to conduct self-examination and visit a dermatologist for regular check-ups to avoid such fatal diseases [11].

### 1.1. Motivation and Contribution

Table 1 exhibits the comparison of the presented survey with further reviews of the skin cancer recognition system. The presented article tries to cover the maximum aspects and contents of the problem domain. The aim of presenting this review is to confer the computer-aided diagnosis methods that are available to recognize skin cancer. Systematic reviews are gathered and existing methods are evaluated according to determined evaluation standards. All the related information is taken from primary sources and after analysis, it gives the details about the utilized methods, their results, and issues encountered by the researchers. The death and death estimation rates in the future due to skin cancer are very high. So, these facts motivate us to contribute in terms of trying to cover the maximum amount of literature for a better understanding of the problem domain and its proposed solutions.

### 1.2. Scope and Objectives

The identification of cancer and its classes with the support of machines can launch and open a new direction in research in an early phase and reduce manual efforts. The rising cases of skin cancer create an alarming situation, so it becomes essential to find cancer at the initial phase to rescue the patient’s life. This research is focused on detecting skin cancer and its classification by reviewing numerous techniques. The entire machine-based processing is performed by image processing, computer vision, and machine learning approaches.

Section 1 includes an introduction along with the scope, objectives, and contribution of the presented research article. Section 2 consists of the number of steps utilized for the skin cancer recognition system. Section 3 includes the number of challenges that occur in the detection process. Section 4 consists of details about the benchmark datasets, Section 5 covers some mobile applications utilized to examine skin cancer through smartphones. Section 6 provides a discussion of methods their encountered problems. Section 7 and its subsection conclude the research article and provide some future suggestion to help researchers contribute to future research.

## 2. Skin Cancer Recognition and Classification System

A computer-aided diagnostic system consists of different phases, these phases are mentioned below:

### 2.1. Preprocessing

It is a very important step when images carry unnecessary details such as hairs, low illumination, spots around the lesion, etc. These elements can degrade the performance of the system. To remove such artifacts [18,19] preprocessing is applied and it also enhances the quality of samples. There are several processes in preprocessing approaches listed below:

#### 2.1.1. Morphological Operations

The artifacts in the skin lesion images can cause the performance to degrade the system. To remove these artifacts many morphological operations are performed. The word morphology defines the outline of the shape and structure of a particular object. These filters are constructed using a set of algebra called mathematical morphology. It is utilized within the window filter hence these are closely related to order statistical filters. In the skin cancer recognition process, various morphological operations are performed to remove the artifacts such as morphology and threshold [20], top-hat approach [21], morphological closing [22,23,24], and morphological operator bottom hat transforms [25], etc.

#### 2.1.2. Colorspace Conversion

Recognizing and discriminating the information in color is considered a major aspect of vision. Color becomes a very useful aspect in the phase of preprocessing, as the color space conversion utilizes a specific function to improve the quality of an image. In skin cancer recognition systems the lesion images are most commonly converted to CIELAB conversion [26,27], HSV color space conversion [28], grayscale [29,30], etc., to improve the visual effect.

#### 2.1.3. Filtering and Other Enhancement Methods

The noise and artifacts are added to the images by several factors during image acquisition and transmission etc. Once the noise is taken out, it makes a great effect on the condition of the image. The noise removal [19,31] algorithm is known as a process that removes and reduces the noise [32] in an image by smoothing the entire image. Image enhancement [33] makes an image lighter or darker or develops slash contrast. It provides a boost to the sensitivity of the information in images and presents enhanced input to carry out other procedures. In skin cancer recognition the research utilized an anisotropic diffusion filter [34,35], median filter [35,36], Dull Razor [37,38,39,40], Adam Huang algorithm [41], Wiener filter [42], Bilateral filter [43] and Gaussian and Gaussian Blur filter [44,45], Z-score transformation [46], contrast-limited adaptive histogram equalization [47,48], adaptive histogram equalization [49], global-local contrast stretching [50], color constancy with shades of gray [51], adaptive gamma correction [52], gamma and correction [53], etc., for enhancement and noise removal in the skin recognition process.

### 2.2. Segmentation

Segmentation [54,55] of skin lesions [56] is the most important step as it divides the single image into various small parts. The researcher proposed several deep learning [57,58] and traditional segmentation [59] methods for skin lesions.

#### 2.2.1. Traditional Segmentation

A hybrid segmentation framework is proposed by researchers, the model combines novel hierarchical k-means with a level set technique [60]. A two-phase segmentation is defined in which firstly the image is segmented with k-means clustering for localization of a precise lesion region then it gets optimized using the firefly algorithm to execute higher accuracy [61]. A histogram-based clustering algorithm is utilized with Genetic algorithm, after that the neutrosophic set are computed by neutrosophic c-mean and at last graph cut is applied for skin lesion [62]. For the segment, region-growing segmentation based on fuzzy clustering segmentation is utilized. The fuzzy clustering mean is unsupervised clustering the extension of k means clustering and region growing Emprise spatial context by merging adjacent pixels [63]. As the homogeneity is determined by texture and color features, researchers utilized color features to partition the image thus segmentation is applied using K-means clustering and histogram calculation [64]. The system described can segment skin images using a semi-supervised approach shift mean algorithm it does not require mentioning the cluster’s numbers [65]. The threshold-based segmentation technique is utilized, as the sample is split into many regions depending on the threshold merit so that the edges of the cancerous area become clear [66]. A skin lesion segmentation technique was designed that established adaptive thresholding with the normalization of color networks for dermoscopic images [67]. The researchers designed an algorithm that solves the problem of global optimization as it established an auxiliary function that was smoothed by utilizing Bezier curves and constructed using a local minimizer [68]. Active contour fusion segmentation is utilized and the main focus is to segment low-contrast dermoscopic samples [50].

#### 2.2.2. CNN-Based Segmentation

An initial contour without the edge chan-vese optimized with the genetic algorithm is proposed for recognition of the skin lesion boundary [69]. For the segmentation in dermoscopic samples, a full convolution encoder-decoder network was optimized exponential neighborhood gray wolf optimization algorithm [70]. The researcher designed a novel CNN-based architecture end-to-end atrous spatial pyramid pooling for segmentation of the lesion [71]. A system designed segments skin lesions with the help of Retina-DeepLab, graph-based techniques, and Mask R-CNN [72]. A dense encoder-decoder-based framework is utilized in which the combination of ResNet and DenseNet is utilized for improvement. Moreover, ASPP is used to get multiscale contextual information and skip connections to recover the information [73]. An automated lesion segmentation using an adaptive dual attention component with three characteristics is proposed in which the first characteristic is two global context modeling schemes integrated with ADAM, the second characteristic is to support the multi-scale fusion for better segmentation and the third is to harness spatial information weighted technique to reduce redundancies [74]. The most effective approach based on an enhanced fully convolutional network approach (iFCN) segments the skin lesion without preprocessing or post-processing. It contributes to the determination of the center position of the lesion and clears the details on the edge by removing the unwanted effects [75]. A method is defined that automatically segments the skin lesion and introduces the novel segmentation topology approach named FC-DPN that is made with the combination of a fully convolutional and dual path network [76]. The researchers proposed an attentive border-aware system for segmentation of multi-scale lesions through adversarial schooling consisting of different sections, ResNet34 as the encoding and decoding path, skip connection based on Scale-Att-ASPP and PPM at the peak of the last convolutional layer in the encoding path [77]. The Mask R-CNN-based technique [78] proposed for skin lesion segmentation consists of two parts, proposing the candidate object bounding boxes with RPN and Fast R-CNN classifier and a branch of binary mask prediction [79]. The researcher proposed effective and novel practice of lesion segmentation by using the fusion of YOLOv3 and the GrabCut algorithm [80]. A lesion segmentation system motivated by the Pyramid Scene Parsing Network in which an encoder-decoder system is designed that uses pyramid pooling blocks, and a skip connection which can pay back for lost spatial details and aggregate global context is utilized [81]. The skin lesion segmentation which locates the lesion accurately with the deep learning method depends on DeepLabv3+ and Mask R-CNN which are utilized to increase the performance [82]. The researchers described the investigation of the relevancy of deep learning by utilizing a pre-trained VGG16 encoder and combined it with DeeplabV3, SegNet decoder, and TernausNet [83]. A deep learning strategy to perform and refine the important task of skin lesion segmentation is utilized by 46 layered U-net frameworks and a modified U-Net framework to achieve a successful lesion segmentation rate [84]. To resolve the challenges of varying size and the appearance of skin lesion segmentation, a new dense deconvolutional framework is designed. The deconvolutional layers are utilized to unchange the dimension of input/output, chained residual pooling extract the contextual background information then fuse multi-level features. Hierarchical supervision is added to make refinements of the prediction mask and serves as auxiliary loss [85].

Table 2 presents a detailed summary of the segmentation methods proposed by researchers. It consists of methods, datasets, and the highest result outcome of the experiment regarding accuracy and Jaccard.

**Figure 1:**
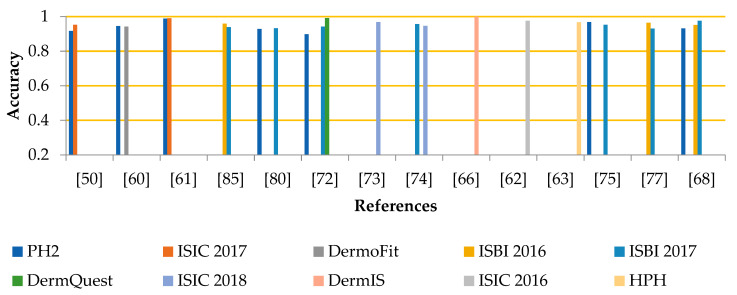
Segmentation Results Concerning the Accuracy [50,60,61,62,63,66,68,72,73,74,75,77,80,85].

**Figure 2:**
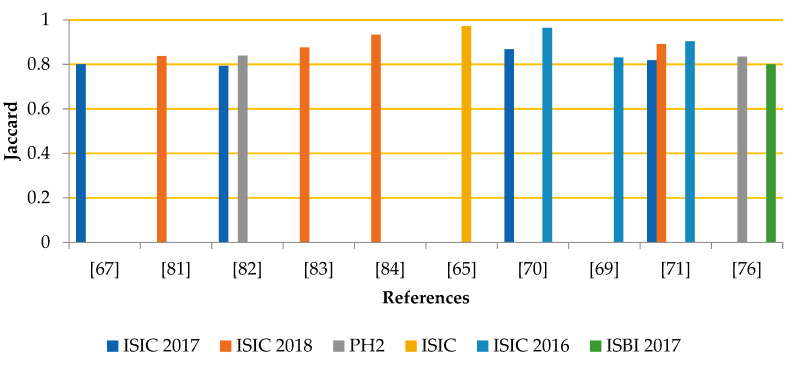
Segmentation Outcomes Concerning the Jaccard [65,67,69,70,71,76,81,82,83,84].

The segmentation experiment is evaluated using different parameters. The graph in Figure 1 shows the comparison among segmentation results in terms of accuracy, while Figure 2 compares segmentation outcomes for Jaccard. However, the process of segmentation is a tough task because of various hurdles such as low illumination and further artifacts. This makes it tricky to identify the exact ROI in the samples. Although researchers designed several algorithms this area is still under development.

### 2.3. Features Extraction, Selection, and Fusion

It is utilized to recognize different features using a variety of machine-learning techniques. It is also the part of dimensionality reduction in which the initial data are first divided and then reduced in a manageable form. The large dataset has several variables that required a lot of computing and some of the variables were also not relevant to the objective. Thus, feature extraction [86] and selection [87,88] help to select the more relevant [89], prominent, and significant features [90,91] and effectively decreased a large mass of variables. In skin cancer recognition, several feature extraction and fusion [92] approaches utilized by researchers are listed below:

#### 2.3.1. Features Extraction

The features were extracted using ABCD, color features were brought out by three-color spaces HSV, LAB, RGB, and texture features by GLCM, after that the most relevant features were selected by a genetic algorithm [93]. In feature extraction, the researchers proposed to refine the bag of words technique by the combination of the features gain from first-order moments and color histogram with HOG [94]. The researchers employed a Local Binary Pattern, Local Vector Pattern, and LTrP that determine eight connections to the neighbors at distance D and also encode them with the help of CST. The combination of LBP+ LVP obtained higher results as compared to other features [95]. The researchers utilized the ABCD method that extracts attributes of symmetry; border, color, and diameter while GLCM and HOG extract the texture features of the lesions in classification SVM provides the best outcomes regarding accuracy [96]. The texture features consider one of the dominant features in the image to obtain second-order texture attributes GLCM of spatial and color features, a color histogram is used to gain color attributes from three color spaces including OPP, HSV, and RGB [97]. The global texture is computed through GLCM and the local features of the sample are taken out using Speeded Up Robust Feature. The comparison shows that SURF features perform better than GLCM and SIFT features and SVM performs better than KNN in classification [98]. The statistical feature standard deviation, mean texture, smoothness, skewness, and energy are extracted by the Local Binary Pattern [99]. In the phase of feature extraction color and textural features combine with a bag of words. Moreover, HL and HG were bagged separately and combined with other bagged Zernike and bagged angles of color vector to emprise the color information [100]. To extract color-based features standard deviation, min, mean, skewness, and kurtosis were calculated for each R,G,B component, and texture-based features were extracted by wavelet transform [101]. The researchers present four ReliefF, Chi2, RFE, and CFS approaches, after that feature normalization is performed using standard score transformation [102].

#### 2.3.2. Features Selection and Fusion

The hybrid features are extracted to classify skin cancer in a machine vision approach. The GLCM and first-order histogram features are combined, moreover, dimensions get reduced with the help of PCA [103]. The researchers extract modified ABCD by using cumulative level difference mean and prominent attributes selected by the Eigenvector centrality feature ranking and selection approach [104]. To recognize melanoma, the network training is performed on the most significant features, the features gained by the GLCM approach, and it gets optimized by a binary bat algorithm which provides a relevant set of features [105]. The novel approach described is called an optimized framework of optimal color feature selections and the best and optimal features get selected using higher entropy value features with PCA [106]. The motive is to distinguish the cancerous and non-cancerous lesions with the help of an accurate feature extraction method so the researchers proposed the fusion of speeded-up-robust features with a bag of features [107].

The researchers proposed several approaches to extract the worthiest features, but to reduce the error rate that occurs due to complex features there is a need to extract more optimal features along with optimization.

### 2.4. Classification

Classification [108,109] is considered the most significant step in skin cancer recognition as it categorizes skin lesions [110]. Firstly, the classifier is trained on some label data after good training, some un-label images are provided to the classifier for testing purposes. There are many machine learning classifiers [111], their variants [112], and CNN [113] models utilized for classification purposes.

#### 2.4.1. Traditional Machine Learning Classifiers

In the domain of skin cancer recognition machine learning [114] performs very well. A method proposed to discover melanoma skin cancer using the ISIC dataset for classification, the researchers utilized an SVM and achieved an accuracy of 96.9% [115]. The researchers utilized different classifiers SVM, KNN, Ensemble, and Decision Tree for melanoma discovery with accuracies of 100%, 87.5%, 87.5%, and 75.0%, respectively [116]. The researchers designed a manner to classify the skin lesion with a decision tree-based Random Forest classifier on ISIC 2017 and HAM 10,000 datasets with an accuracy of 97% [117]. The Naïve Bayes classifier was utilized for dermoscopic classification using the Dermatology Information System and DermQuest with an accuracy of 98.8% [118]. The researchers utilized SVM, KNN, Ensemble classifiers, and Artificial Neural Network classifier and their variants. The SVM variant gives the best accuracy of 83% [119]. For melanoma, keratosis, and benign discovery the Naïve Bayes give accuracies of 91.2%, 92.9%, and 94.3%, respectively [120]. A system was proposed based on fuzzy decision ontology for the recognition of melanoma with the help of a KNN classifier on DermQuest and Dermatology Information System with an accuracy of 92% [121]. The researchers designed a system for the acknowledgment of melanoma with ANN and SVM classifiers which obtained an accuracy of 96.2% and 97%, respectively [122].

#### 2.4.2. Deep Learning Models

Deep learning [123] is strengthening its roots in every research domain nowadays. Various advanced and deep learning [124,125] building blocks are employed by researchers to acquire worthy performances. A stacked ensemble framework based on CNN is designed to recognize melanoma in the initial stage, in which the multiple sub-CNN models for classification tasks are an ensemble. A task is performed on the ISIC dataset having two classes with an accuracy of 0.957 [126]. The researchers utilized three models VGG16, VGG19, and Inception V3 using the ISIC and obtained accuracies of 77%, 76%, and 74%, respectively [127]. A methodology is defined that classifies samples of skin cancer with the help of ResNet, VGG19, and InceptionV3 on more than 24,000 samples. Moreover, experiments show that inceptionV3 performs best [128]. The classification of skin lesions was performed with EfficientNets and ensemble models using the ISIC 2020 melanoma classification dataset, the best result is recorded using the entire EfficientNet B6 models ensemble and one is EfficientNet B5 with a 0.944 ROC curve [129]. A CNN architecture was utilized for pigmented lesion categorization using the HAM10000 dataset with an accuracy of 91.51% [130]. The VGG 16 and AlexNet were utilized which were serially fused and optimized using PCA. The classification was performed using PH2, ISBI 2016, and 2017 datasets with an accuracy of 99% [131]. A system was proposed that distinguishes different classes of skin cancer through five pre-trained CNN and four ensemble models on the HAM-10000 dataset in which pre-trained ResNetXt101 and ensemble InceptionResNetV2+ ResNetXt101 provide the best accuracies of 93.20% and 92.83%, respectively [132]. The GoogleNet model performed classification using the ISIC 2019 with an accuracy of 94.92% [133]. The researcher designed a hyper-connected CNN called HcCNN for the discrimination of skin lesions in a multimodality test on a seven point checklist and achieved an accuracy of 74.9% [134]. The classification using CNN with a Novel Regularizer is proposed which is a binary approach that distinguishes malignant and benign lesions tested on ISIC and provides an accuracy of 97.49% [135]. The ensemble of CNN networks for classification for melanoma recognition including InceptionV4, SENet154, InceptionResNetV2, and PNASNeT-5-Large was utilized, in which PNASNeT-5-Large gives an excessive validation outcome that is 0.76 on the ISIC 2018 dataset [136]. The insufficient training data problem gets resolved by intra-class difference and inter-class alikeness by designing the ARL-CNN using ISIC 2017 with an accuracy of 85% [137]. The CNN framework MobileNet proposed for the categorization of skin lesions using the HAM-10000 dataset in which accuracy is a little bit higher without data augmentation is 83.93% [138]. The GoogLleNet, VGG, and their ensemble were utilized for the categorization of seven classes utilizing the ISIC 2018 and the models are 79.7%, 80.1% and 81.5% accurate, respectively [139]. The summary of the existing classification approaches along with highest obtained results are mentioned in Table 3.

**Figure 3:**
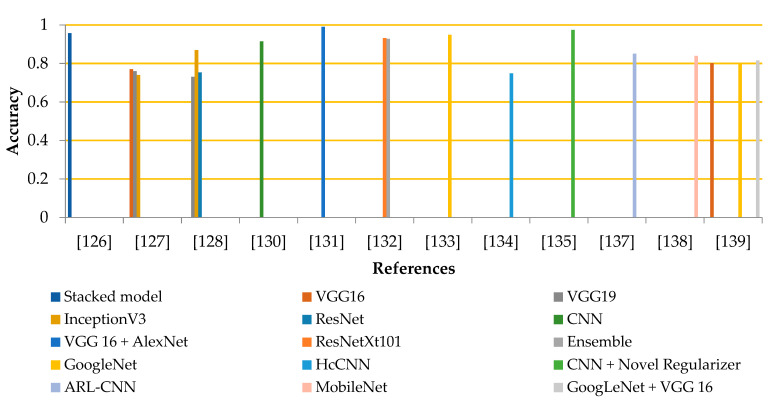
Graphical Comparative Analysis of Classification Outcomes [126,127,128,130,131,132,133,134,135,137,138,139].

In the classification process, the problem of less training data, overfitting, and underfitting of the model cause misclassification and make the wrong prediction. Figure 3 presents a visual representation of the outcomes of the CNN classification.

## 3. Challenges in the Existing Literature

The processing task on skin-infected images is very challenging due to several reasons. The most important reason is low contrast which makes it difficult to differentiate the infected and normal skin. Other factors include the presence of hair, bubble, ruler, and ink artifacts. So it is important to consider these artifacts while making algorithms [140]. The other challenges include extensive training, images of light-skinned persons in standard datasets, small interclass variation, multi-sized and multi-shape images, and unbalanced classes of datasets.

## 4. Benchmark Datasets

Following is the list of standard available datasets: HAM-10000: The dataset contains seven classes of skin lesions. The dermatoscopic images are stored in different modalities. The total number of samples are 10,015 which are publicly obtained through the ISIC archive [141]. MED–NODE: The dataset contains 170 samples [142]. PH2: It consists of 200 dermoscopic images [143] with three classes of normal, abnormal, and melanoma [144]. DermoFit: The DermoFit consists of macroscopic images [145] and the repository contains 1300 images with 10 classes with parallel binary segmentation masks [146]. ISBI 2016: The dataset is provided by the ISIC archive. The whole count of samples in the dataset is 1279 of which 900 are training and 379 are testing images [147]. ISBI 2017: It contains a total of 2750 images, out of which 150 are for validation, 2000 for training, and 600 for testing. [148]. ISIC 2018: The ISIC is the world’s largest database that provides 12,500 images with three tasks. The first task is lesion segmentation, the second task is the detection of lesion attributes, and the last task is a classification of lesion disease. The dataset consists of seven classes [149]. ISIC 2019: The data in ISIC 2019 come from HAM-10000, BCN_20000, ViDIR Group, and anonymous resources. The dataset consists of 25,331 dermoscopic samples with eight classes [150]. ISIC 2020: The dataset consists of 33,126 samples. The dataset presents two formats that are DICOM, where pixels data are encoded with JPEG [151].

## 5. Mobile Apps for Skin Cancer Detection

The users can perform a self-examination of skin lesions and determine the risk of melanoma by taking a picture from a smartphone. There are several applications some of which are discussed below: Mole Mapper: the application collects the data provided by the participant and measurements of the mole with behavioral and demographic information that is related to the risk of melanoma. The application should be downloaded from the Apple App store [152]. m-Skin Doctor: utilizing image processing and computer vision approaches, the researchers described a real-time mobile healthcare system that can recognize melanoma. The Gaussian removes noise and segmentation is undertaken by the GrabCut algorithm. In the last step, SVM performs the classification. The application provides a specificity of 75% and a sensitivity of 80% [153]. SkinVision: this is a smartphone application with 900,000 users worldwide. There are different versions of the app, and the latest version was launched in October 2018 which is the fifth version. The app uses a conditional generative adversarial neural network and SVM. It obtains 78% specificity and 95% sensitivity [154]. UMSkinCheck: the application was developed by the University of Michigan. The full-body survey required the taking of 23 photographs in seven positions for future lesion comparison. The risk calculator discovers the risk of the development of melanoma built on the ten previously detected risk factors [155]. SpotMole: it is an android application that allows the direct submission of an image captured using the app and in-direct submission using the gallery. The application provides a specificity of 80% and a sensitivity of 43% [156].

## 6. Discussion

According to the facts, melanoma has become among the most rapidly increasing cancers all around the globe, but it can be cured if detected at its initial stage. It should be noted that many state-of-art-techniques consist of various problems, which affect their achievements and generate faulty outcomes while recognizing skin cancer. This research article presented a comprehensive literature review on automated skin cancer identification processes with deep learning and machine learning approaches. Different approaches were analyzed and compared on benchmark datasets. However, issues such as noise, poor contrast, border irregularity, etc. [126] reduced the effectiveness of the approaches, so the algorithms must be able to handle these hurdles. Existing standard datasets contain samples of light-skinned persons [157], datasets having enough images of both light and dark-skinned people are necessary to increase the accuracy of skin cancer recognition systems. Sometimes classes of datasets are unbalanced [138] and due to the problem of deficient training data, so the dataset must be augmented to balance each class, to draw strong generalizations.

## 7. Conclusions

The examination of a lesion by the naked eye is not accurate and the medical procedure of skin cancer diagnosis is reliable, but it takes lots of time. Accurate lesion segmentation in dermoscopy samples is an important and difficult job. Thus, a computer-aided diagnosis system is utilized to perform these tasks more accurately. This comprehensive survey article has discussed several noninvasive machine and deep learning approaches for skin cancer recognition and classification. Multiple steps required for the skin cancer recognition system, including preprocessing and segmentation go along with feature extraction and classification. This survey focused on the latest approaches designed for the identification of skin cancer. Every algorithm has its own merits and limitations so that specific selection of algorithms according to the problem is the best outcome. However, CNN provides better outcomes than other traditional approaches while classifying image samples and segmentation because it is more interconnected to computer vision than others. This article provides extensive literature on the techniques utilized for the skin cancer detection process.

### Future Direction

In the future, researchers may survey skin cancer detection systems by more categorization in sections. Traditional segmentation is further divided into region-based, clustering-based, threshold-based, etc. Similarly, the feature extraction should be categorized into color features, shape features, and texture features. Although the results obtained from CNN are impressive still, some limitations such as optimization and generalization exist which could be solved in the future using Quantum computing.

## Figures and Tables

**Table 1:** Comparison of the Presented Survey with Others.

Sr. #	Contents	Presented Survey	[12] 2021	[13] 2020	[14] 2020	[15] 2020	[16] 2019	[17] 2017
1.	Traditional methods	✓	✓	✓	✓	✓	✓	✓
2.	Deep learning methods	✓	✓	✗	✓	✓	✓	✗
3.	Benchmark datasets	✓	✓	✓	✗	✓	✗	✗
4.	Challenges	✓	✗	✓	✗	✓	✓	✗
5.	Mobile Apps	✓	✗	✗	✗	✗	✗	✗
6.	Discussion/Findings	✓	✓	✗	✗	✓	✓	✗

**Table 2:** Summary of Segmentation Approaches.

Ref #	Year	Methods	Datasets	Results
[50]	2019	Local and global contrast starching, Dull razor, Region based active contour JSEG fusion	PH2 ISIC 2017	0.917 (ACC) 0.953 (ACC)
[60]	2021	Histogram equalization, max filter, morphological operations, hierarchical k-means with level set	PH2 DermoFit	0.946 (ACC) 0.942 (ACC)
[61]	2021	Threshold and morphological operations, K-means clustering, firefly algorithm	ISIC 2017 PH2	0.991 (ACC) 0.989 (ACC)
[85]	2018	Adam approach, a Dense deconvolutional network	ISBI 2016 ISBI 2017	0.959 (ACC) 0.939 (ACC)
[80]	2019	Dull razor, morphological operations, YOLO, GrabCut	PH2 ISBI 2017	0.929 (ACC) 0.933 (ACC)
[72]	2021	Retina-DeepLab, R-CNN, and graph-related approaches	ISBI 2017 PH2 DermQuest	0.942 (ACC) 0.898 (ACC) 0.992 (ACC)
[73]	2021	Data augmentation (flipping, rotation, translation, etc.), dense encoder-decoder based framework	ISIC 2018	0.969 (ACC)
[74]	2020	Bicubic interpolation, data augmentation, Adaptive dual attention module	ISBI 2017 ISIC 2018	0.957 (ACC) 0.947 (ACC)
[68]	2020	Median filter, histogram, Auxiliary function, global optimization algorithm	PH2 ISBI 2016 ISBI 2017	0.932 (ACC) 0.952 (ACC) 0.976 (ACC)
[66]	2020	Median filter, contrast stretching, ABCD, Threshold-based segmentation	DermIS DermQuest	1.00 (ACC)
[62]	2020	GA, OCE-NGC	ISIC 2016	0.976 (ACC)
[63]	2017	Fuzzy clustering mean	HPH	0.968 (ACC)
[75]	2020	Ifcn	PH2 ISBI 2017	0.969 (ACC) 0.953 (ACC)
[77]	2020	Resizing, augmentation, ResNet34, Scale-Att-ASPP, PPM, GAN	ISBI 2016 ISBI 2017 PH2	0.964 (ACC) 0.931 (ACC) 0.112 (DV)
[79]	2020	LabelMe, R-CNN	ISIC	0.910 (recall)
[67]	2019	Gaussian filter, OTSU, SegRNorm, SegXNorm	ISIC 2017	0.800 (JAC)
[81]	2019	Resizing, encoder-decoder deep convolutional with aggregate multi-resolution skip connections	ISIC 2018	0.837 (JAC)
[82]	2019	Morphological operations, DeeplabV3+ and Mask R-CNN	ISIC 2017 PH2	0.793 (JAC) 0.839 (JAC)
[83]	2019	Data augmentation, VGG16 encoder, DeeplabV3, SegNet, threshold with dilations	ISIC 2018	0.876 (JAC)
[84]	2019	Linear filter, restoration, enhancement, U-Net 46 layered, U-Net 32 layered	ISIC 2018	0.933 (JAC)
[64]	2017	Illumination correction, histogram calculation, Frangi vesselness, K- means clustering	ISIC 2017	0.548 (validation set)
[65]	2017	Dull razor, ABCD, Shift mean algorithm	ISIC	0.972 (JAC)
[70]	2022	Image Resize, FCEDN, EN-GWO	ISIC 2016 17	0. 964 (JAC) 0.868 (JAC)
[69]	2021	Dull razor, Initial contour optimization, GA	ISIC 2016	0.831 (JAC)
[71]	2021	Downsampling, translation, rotation and scaling, Atrous dilation CNN	ISIC 2016 17 18	0.904 (JAC) 0.818 (JAC) 0.891 (JAC)
[76]	2020	Augmentation, resizing, FC-DPN	ISBI 2017 PH2	0.800 (JAC) 0.835 (JAC)

**Table 3:** Summary of Classification Techniques.

Ref #	Year	CNN Models	Datasets	No. of Classes	Results (ACC)
[126]	2022	Stacked model	ISIC 2020	2	0.957
[127]	2021	VGG16, VGG19, InceptionV3	ISIC 2018	7	0.770 0.760 0.740
[128]	2021	ResNet, InceptionV3, VGG19	ISIC archive	2	0.753 0.869 0.731
[129]	2021	EfficientNet B6 models ensemble and EfficientNet B5	ISIC 2020	2	0.941(ROC curve)
[130]	2021	CNN architecture	HAM-10000	7	0.915
[131]	2020	VGG 16 + AlexNet	PH2+ +ISBI 2016 +ISBI 2017	2	0.990
[132]	2020	ResNetXt101, InceptionResNetV2+ ResNetXt101	HAM-10000	7	0.932 0.928
[133]	2020	GoogleNet	ISIC 2019	8	0.949 0.798(SEN) 0.970 (SPE)
[134]	2020	HcCNN	7 point check	7	0.749
[135]	2019	CNN + Novel Regularizer	ISIC archive	2	0.974
[136]	2019	PNASNeT-5-Large	ISIC 2018	7	0.76 (V.score)
[137]	2019	ARL-CNN	ISIC 2017	3	0.850
[138]	2019	MobileNet	HAM-10000	7	0.839
[139]	2018	GoogleNet, VGG 16, and their ensemble	ISIC 2018	7	0.797 0.801 0.815
