# Correction: Zafar et al. Skin Lesion Analysis and Cancer Detection Based on Machine/Deep Learning Techniques: A Comprehensive Survey. *Life* 2023, *13*, 146

**DOI:** 10.3390/life15020316

**Published:** 2025-02-18

**Authors:** Mehwish Zafar, Muhammad Imran Sharif, Muhammad Irfan Sharif, Seifedine Kadry, Syed Ahmad Chan Bukhari, Hafiz Tayyab Rauf

**Affiliations:** 1 Department of Computer Science, COMSATS University Islamabad, Wah Campus, Wah Cantt 47040, Pakistan; mehvishzafar999@gmail.com (M.Z.);; 2 Department of Computer Science, University of Education, Jauharabad Campus, Khushāb 41200, Pakistan; muhammadirfaansharif@gmail.com; 3 Department of Applied Data Science, Noroff University College, 4612 Kristiansand, Norway; 4 Artificial Intelligence Research Center (AIRC), Ajman University, Ajman P.O. Box 346, United Arab Emirates; 5 Department of Electrical and Computer Engineering, Lebanese American University, Byblos P.O. Box 13-5053, Lebanon; 6 Division of Computer Science, Mathematics and Science, Collins College of Professional Studies, St. John’s University, Queens, NY 11439, USA; bukharis@stjohns.edu; 7 Independent Researcher, Bradford BD8 0HS, UK; hafiztayyabrauf093@gmail.com

## Corrected Affiliation

In the published publication [1], there was an error regarding the affiliation for Hafiz Tayyab Rauf. The updated affiliation should be as follows: Independent Researcher, Bradford BD8 0HS, UK. The authors state that the scientific conclusions are unaffected. This correction was approved by the Academic Editor. The original publication has also been updated.
